# Twenty-Year Trends in Diagnosed Attention-Deficit/Hyperactivity Disorder Among US Children and Adolescents, 1997-2016

**DOI:** 10.1001/jamanetworkopen.2018.1471

**Published:** 2018-08-31

**Authors:** Guifeng Xu, Lane Strathearn, Buyun Liu, Binrang Yang, Wei Bao

**Affiliations:** 1 Center for Disabilities and Development, University of Iowa Stead Family Children’s Hospital, Iowa City; 2 Department of Epidemiology, College of Public Health, University of Iowa, Iowa City; 3 Division of Developmental and Behavioral Pediatrics, Stead Family Department of Pediatrics, University of Iowa Carver College of Medicine, Iowa City; 4 Department of Developmental and Behavioral Pediatrics, Shenzhen Children’s Hospital, Shenzhen, Guangdong, China

## Abstract

**Importance:**

Attention-deficit/hyperactivity disorder (ADHD) is common in US children and adolescents. It is important to understand the most recent prevalence of ADHD and its long-term trends over the past decades.

**Objective:**

To estimate the prevalence of diagnosed ADHD and 20-year trends from 1997 to 2016 among US children and adolescents using nationally representative data.

**Design, Setting, and Participants:**

In this population-based, cross-sectional survey study (National Health Interview Survey), surveys were conducted annually from 1997 to 2016. A total of 186 457 children and adolescents aged 4 to 17 years from 1997 to 2016 were included in this analysis. Data were collected through in-person household interviews with a parent or guardian. The data analysis was performed in January 2018.

**Main Outcomes and Measures:**

Attention-deficit/hyperactivity disorder diagnosed by a physician or other health care professional.

**Results:**

Among the included 186 457 children and adolescents (96 017 boys [51.5%], 51 350 Hispanic [27.5%], 91 374 non-Hispanic white [49.0%], 28 808 non-Hispanic black [15.5%], 14 925 non-Hispanic other race [8.0%]), 14 704 children and adolescents (7.9%; 10 536 boys [71.7%], 2497 Hispanic [17.0%], 9010 non-Hispanic white [61.3%], 2328 non-Hispanic black [15.8%], and 869 non-Hispanic other race [5.9%]) were reported to have ever been diagnosed with ADHD. The weighted prevalence of diagnosed ADHD was 10.2% (95% CI, 9.6%-10.8%) in 2015-2016. There were significant sex and racial/ethnic disparities in the prevalence of diagnosed ADHD. The prevalence was 14.0% (95% CI, 13.1%-15.0%) in boys and 6.3% (95% CI, 5.6%-7.0%) in girls, 6.1% (95% CI, 5.2%-7.0%) in Hispanic individuals, 12.0% (95% CI, 11.1%-12.9%) in non-Hispanic white individuals, and 12.8% (95% CI, 11.0%-14.5%) in non-Hispanic black individuals. Over the 20-year period, the estimated prevalence of diagnosed ADHD in US children and adolescents increased from 6.1% in 1997-1998 to 10.2% in 2015-2016 (*P* for trend <.001). All subgroups by age, sex, race/ethnicity, family income, and geographic regions showed a significant increase in the prevalence from 1997-1998 to 2015-2016.

**Conclusions and Relevance:**

This study’s findings suggest that among US children and adolescents, the estimated prevalence of diagnosed ADHD increased significantly between 1997-1998 and 2015-2016. This study suggests that additional research is needed to better understand the cause of this apparent rise in prevalence.

## Key Points

**Question:**

What are the long-term trends in prevalence of attention-deficit/hyperactivity disorder among US children and adolescents over the past 2 decades?

**Findings:**

In this study of data from 186 457 children and adolescents aged 4 to 17 years from the National Health Interview Survey, a nationwide, population-based, cross-sectional survey conducted annually from 1997 to 2016, the estimated prevalence of diagnosed attention-deficit/hyperactivity disorder in US children and adolescents increased from 6.1% in 1997-1998 to 10.2% in 2015-2016.

**Meaning:**

Among US children and adolescents, the estimated prevalence of diagnosed attention-deficit/hyperactivity disorder increased significantly between 1997 and 2016.

## Teaser

This survey study uses data from the National Health Interview Survey to examine changes in the prevalence of diagnosed attention-deficit/hyperactivity disorder (ADHD) in US children and adolescents over a 20-year period, from 1997 to 2016.

## Introduction

Attention-deficit/hyperactivity disorder (ADHD) is a childhood-onset neuropsychiatric disorder characterized by persistent and impairing inattention, hyperactivity, and impulsivity.^1,2^ The symptoms of ADHD often persist into adulthood. Early comorbidities concurrent with ADHD may include tic disorder, anxiety disorder, autism spectrum disorder, communication and specific learning or motor disorders (eg, reading disability, developmental coordination disorder), and intellectual disability.^1^ Long-term follow-up studies from childhood to adulthood found that children with ADHD, compared with those without ADHD, were more impaired in psychosocial, educational, and neuropsychological functioning^3^ and had higher risks for antisocial disorders, major depression, and anxiety disorders as adults.^4^

The American Psychiatric Association states in the *Diagnostic and Statistical Manual of Mental Disorders, Fifth Edition* that 5% of children have ADHD, based on previous worldwide estimates in earlier years.^5,6^ The prevalence of ADHD varies across different countries, with a significantly higher prevalence in the United States than in European countries.^7^ Moreover, the prevalence of ADHD has changed over time. Several previous studies in the United States have shown an increase in ADHD prevalence over the past years.^8,9,10,11,12,13,14,15,16,17,18,19^ For example, an analysis of the National Health Interview Survey (NHIS) reported a 33% increase in ADHD prevalence from 1997-1999 (5.7%) to 2006-2008 (7.6%) among children and adolescents aged 3 to 17 years.^8^ Similarly, the National Survey of Children’s Health showed a 42% increase between 2003 and 2011 in the prevalence of diagnosed ADHD among children and adolescents aged 4 to 17 years.^9^

Information about the current prevalence of ADHD and its long-term trends over the past decades is needed to inform future research, clinical care, and policy decision making on ADHD. Therefore, we analyzed nationally representative data to estimate the most recent prevalence of ADHD diagnosis among US children and adolescents and the 20-year trends from 1997 to 2016.

## Methods

### Study Population

The NHIS is a leading national health survey in the United States.^20^ It is conducted annually by the National Center for Health Statistics at the Centers for Disease Control and Prevention. The NHIS is composed of a series of nationally representative cross-sectional surveys. With a large sample size and a relatively high response rate, the NHIS has become the principal source of information on the health of the civilian, noninstitutionalized household population of the United States.^21^ The NHIS survey methodologic reporting is consistent with the reporting standards by the American Association for Public Opinion Research.^22^ Annual sample size of the NHIS is about 35 000 households containing approximately 87 500 persons. In NHIS 1997-2016, the total household response rate ranged from 67.9% to 91.8% and conditional response rate for the sample child component ranged from 85.6% to 93.3%. The NHIS survey was approved by the National Center for Health Statistics Research Ethics Review Board. All respondents provided informed informed verbal consent prior to participation. The University of Iowa Institutional Review Board determined the present study was exempt from approval given the use of deidentified data. This study followed the American Association for Public Opinion Research (AAPOR) reporting guideline.

### Data Collection

The NHIS collected data on a variety of health topics through in-person household interviews. For each interviewed family in the household, 1 sample child, if any, was randomly selected by a computer program.^20^ Information about the sample child was collected by interviewing an adult, usually a parent, who was knowledgeable about the child's health.

From 1997 to 2016, respondents were asked, “Has a doctor or health professional ever told you that [the sample child] had attention-deficit/hyperactivity disorder (ADHD) or attention-deficit disorder (ADD)?” Approximately 99.8% of the participants responded to this question. The respondents in the 2016 survey were further asked, “Does [the sample child] currently have attention-deficit/hyperactivity disorder (ADHD) or attention-deficit disorder (ADD)?” Demographic data, such as age, sex, race/ethnicity, family income, and geographic region, were collected using a standardized questionnaire during the interview. Race and Hispanic ethnicity were self-reported and classified based on the 1997 Office of Management and Budget Standards. Family income levels were classified according to the ratio of family income to federal poverty level lower than 1.0, 1.0 to 1.9, 2.0 to 3.9, and 4.0 or higher. Data analysis was performed in January 2018.

### Statistical Analysis

All eligible children and adolescents aged 4 to 17 years who participated in the NHIS were included in the present study. Only 0.2% of participants had missing information on ADHD diagnosis and were therefore excluded. We restricted the age range to 4 to 17 years because the clinical guidelines of the American Academy of Pediatrics recommended to initiate an evaluation for ADHD in children aged 4 years or older.^23^

We estimated the prevalence estimates with survey weights to account for unequal probabilities of selection, oversampling, and nonresponse in the survey. We also used survey design variables about strata and primary sampling units for each survey cycle. In this study, we combined each 2-year period (eg, 1997 and 1998) as 1 survey cycle (eg, cycle 1997-1998). *P* values for overall differences across strata were calculated using χ^2^ tests. Trends in the prevalence over time were tested using a weighted logistic regression model, which included survey cycle as a continuous variable and adjusted for age, sex, and race/ethnicity. To determine whether the secular trends differ across strata, interaction analyses were performed by including multiplicative terms of each strata variable with survey cycle in the aforementioned logistic regression models. All analyses were conducted using survey procedures in SAS, version 9.4 (SAS Institute). Two-sided *P* < .05 was considered statistically significant.

## Results

### Prevalence of Diagnosed ADHD Among US Children and Adolescents in 2015-2016

Among the included 18 107 children and adolescents aged 4 to 17 years (9373 boys [51.8%], 4620 Hispanic [25.5%], 9117 non-Hispanic white [50.4%], 2294 non-Hispanic black [12.7%], and 2076 non-Hispanic other race [11.5%]) in 2015-2016, 1880 children and adolescents (1314 boys [69.9%], 286 Hispanic [15.2%], 1143 non-Hispanic white [60.8%], 290 non-Hispanic black [15.4%], and 161 non-Hispanic other race [8.6%]) were reported to have ever been diagnosed with ADHD. The weighted prevalence of ADHD diagnosis was 10.2% (95% CI, 9.6%-10.8%) in 2015-2016 (Table 1). There were significant differences in the prevalence according to age, sex, race/ethnicity, family income, and geographic region. The prevalence was 7.7% (95% CI, 7.0%-8.5%) in children aged 4 to 11 years, 13.5% (95% CI, 12.6%-14.5%) in adolescents aged 12 to 17 years, 14.0% (95% CI, 13.1%-15.0%) in boys, 6.3% (95% CI, 5.6%-7.0%) in girls, 6.1% (95% CI, 5.2%-7.0%) in Hispanic individuals, 12.0% (95% CI, 11.1%-12.9%) in non-Hispanic white individuals, 12.8% (95% CI, 11.0%-14.5%) in non-Hispanic black individuals, 10.3% (95% CI, 8.8%-11.8%) in those living in the Northeast, 12.2% (95% CI, 10.8%-13.6%) of those in the Midwest, 11.1% (95% CI, 10.1%-12.1%) of respondents in the South, and 7.0% (95% CI, 6.1%-7.8%) of those in the West (Table 1).

**Table 1.** Prevalence of Diagnosed ADHD in US Children and Adolescents Aged 4 to 17 Years, 2015-2016

Characteristic	Participants, No. (%)^a^		ADHD Prevalence, % (95% CI)^b^	*P* Value^c^
	Overall	With ADHD		
Overall	18 107	1880	10.2 (9.6-10.8)	
Age, y				
4-11	9735 (53.8)	765 (40.7)	7.7 (7.0-8.5)	<.001
12-17	8372 (46.2)	1115 (59.3)	13.5 (12.6-14.5)	
Sex				
Boys	9373 (51.8)	1314 (68.9)	14.0 (13.1-15.0)	<.001
Girls	8734 (48.2)	566 (30.1)	6.3 (5.6-7.0)	
Race/ethnicity				
Hispanic	4620 (25.5)	286 (15.2)	6.1 (5.2-7.0)	<.001
Non-Hispanic white	9117 (50.4)	1143 (60.8)	12.0 (11.1-12.9)	
Non-Hispanic black	2294 (12.7)	290 (15.4)	12.8 (11.0-14.5)	
Other	2076 (11.5)	161 (8.6)	7.7 (6.0-9.4)	
Family income to poverty ratio				
<1.0	2595 (14.3)	330 (17.6)	12.9 (11.2-14.6)	.004
1.0-1.9	3494 (19.3)	386 (20.5)	10.2 (9.0-11.5)	
2.0-3.9	4496 (24.8)	461 (24.5)	10.0 (8.6-11.3)	
≥4.0	4675 (25.8)	450 (23.9)	9.4 (8.4-10.4)	
Missing	2847 (15.7)	253 (13.5)	9.4 (8.0-10.8)	
Geographic region				
Northeast	2882 (15.9)	341 (18.1)	10.3 (8.8-11.8)	<.001
Midwest	3614 (20.0)	417 (22.2)	12.2 (10.8-13.6)	
South	6375 (35.2)	738 (39.3)	11.1 (10.1-12.1)	
West	5236 (28.9)	384 (20.4)	7.0 (6.1-7.8)	

Abbreviation: ADHD, attention-deficit/hyperactivity disorder.

^a^ The numbers of participants overall and with ADHD were unweighted.

^b^ Prevalence estimates were weighted.

^c^ *P* values for overall differences in prevalence by stratum.

### Trends in Diagnosed ADHD in US Children and Adolescents From 1997 to 2016

To estimate the 20-year trends in diagnosed ADHD from 1997 to 2016, we included 186 457 children and adolescents aged 4 to 17 years (96 017 boys [51.5%], 51 350 Hispanic [27.5%], 91 374 non-Hispanic white [49.0%], 28 808 non-Hispanic black [15.5%], and 14 925 non-Hispanic other race [8.0%]). Among them, 14 704 children and adolescents (7.9%; 10 536 boys [71.7%], 2497 Hispanic [17.0%], 9010 non-Hispanic white [61.3%], 2328 non-Hispanic black [15.8%], and 869 non-Hispanic other race [5.9%]) were reported to have ever been diagnosed with ADHD.

The mean age of the participants over the period was almost identical, ranging from 10.6 to 10.9 years. The estimated prevalence of diagnosed ADHD increased from 6.1% in 1997-1998 to 10.2% in 2015-2016 (*P* for trend <.001) (Figure). All subgroups evaluated showed a significant increase in the prevalence from 1997-1998 to 2015-2016 (Table 2).

**Figure.**
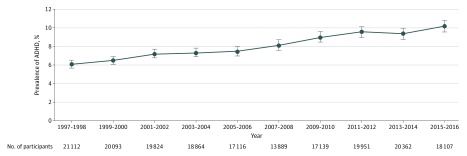
Prevalence of Diagnosed Attention-Deficit/Hyperactivity Disorder (ADHD) in US Children and Adolescents, 1997-2016 Prevalence estimates with 95% CIs (error bars) were weighted. *P* < .001 for trend was calculated using a weighted logistic regression model, which included survey cycle as a continuous variable and adjusted for age, sex, and race/ethnicity.

**Table 2.** Trends in the Prevalence of Diagnosed ADHD in US Children and Adolescents Aged 4 to 17 Years, 1997-2016

Characteristic	ADHD Prevalence, % (95% CI)^a^										*P* Value	
	1997-1998	1999-2000	2001-2002	2003-2004	2005-2006	2007-2008	2009-2010	2011-2012	2013-2014	2015-2016	Trend^b^	Interaction^c^
No. of participants overall^d^	21 112	20 093	19 824	18 864	17 116	13 889	17 139	19 951	20 362	18 107		
No. of participants with ADHD^d^	1243	1264	1387	1360	1235	1107	1470	1857	1901	1880		
Overall prevalence	6.1 (5.7-6.5)	6.5 (6.1-6.9)	7.2 (6.8-7.7)	7.3 (6.9-7.8)	7.5 (7.0-8.0)	8.1 (7.6-8.7)	9.0 (8.5-9.6)	9.6 (9.0-10.1)	9.4 (8.8-9.9)	10.2 (9.6-10.8)	<.001	
Age, y												
4-11	5.3 (4.9-5.8)	5.3 (4.8-5.7)	5.9 (5.4-6.5)	5.8 (5.2-6.4)	6.0 (5.4-6.6)	6.1 (5.5-6.7)	6.9 (6.2-7.5)	7.7 (7.1-8.4)	7.9 (7.2-8.6)	7.7 (7.0-8.5)	<.001	.004
12-17	7.2 (6.6-7.8)	8.2 (7.5-8.9)	8.9 (8.2-9.7)	9.3 (8.6-10.0)	9.3 (8.4-10.2)	10.8 (9.8-11.8)	11.9 (10.9-12.9)	12.0 (11.1-12.9)	11.3 (10.4-12.3)	13.5 (12.6-14.5)	<.001	
Sex												
Boys	9.0 (8.4-9.6)	9.4 (8.8-10.1)	10.3 (9.6-11.1)	10.2 (9.5-10.9)	10.6 (9.7-11.5)	11.3 (10.4-12.2)	12.2 (11.4-13.0)	13.6 (12.7-14.4)	12.8 (12.0-13.7)	14.0 (13.1-15.0)	<.001	<.001
Girls	3.1 (2.7-3.5)	3.4 (2.9-3.8)	4.0 (3.5-4.4)	4.3 (3.8-4.9)	4.2 (3.7-4.7)	4.8 (4.1-5.5)	5.7 (5.1-6.4)	5.4 (4.8-6.0)	5.8 (5.2-6.4)	6.3 (5.6-7.0)	<.001	
Race/ethnicity												
Hispanic	3.6 (2.9-4.3)	3.4 (2.8-4.0)	3.8 (3.2-4.5)	4.1 (3.5-4.7)	5.1 (4.2-6.0)	4.4 (3.6-5.2)	4.8 (4.2-5.5)	6.1 (5.3-6.9)	6.1 (5.4-6.8)	6.1 (5.2-7.0)	<.001	<.001
Non-Hispanic white	7.2 (6.7-7.7)	7.8 (7.2-8.4)	8.4 (7.8-9.1)	8.6 (7.9-9.2)	8.5 (7.7-9.2)	9.6 (8.7-10.5)	10.6 (9.7-11.4)	11.6 (10.8-12.4)	11.4 (10.6-12.2)	12.0 (11.1-12.9)	<.001	
Non-Hispanic black	4.7 (3.9-5.5)	4.8 (4.0-5.6)	7.2 (6.1-8.3)	7.3 (5.9-8.6)	7.5 (6.3-8.8)	8.5 (7.2-9.9)	11.1 (9.7-12.5)	9.4 (8.1-10.7)	8.8 (7.6-10.0)	12.8 (11.0-14.5)	<.001	
Other	3.9 (2.6-5.2)	4.0 (2.7-5.3)	3.7 (2.4-5.0)	5.2 (3.6-6.7)	5.1 (3.7-6.5)	6.0 (4.2-7.9)	6.2 (4.6-7.7)	6.9 (5.3-8.5)	6.9 (5.5-8.4)	7.7 (6.0-9.4)	<.001	
Family income to poverty ratio												
<1.0	6.6 (5.5-7.6)	7.9 (6.5-9.3)	8.9 (7.6-10.3)	7.7 (6.3-9.0)	9.4 (7.9-10.9)	10.0 (8.2-11.8)	12.5 (11.0-14.1)	12.5 (11.0-14.1)	12.0 (10.5-13.5)	12.9 (11.2-14.6)	<.001	<.001
1.0-1.9	7.1 (6.1-8.1)	6.9 (5.9-8.0)	7.3 (6.2-8.4)	7.4 (6.4-8.4)	8.7 (7.3-10.1)	10.0 (8.3-11.7)	10.3 (8.7-11.8)	9.0 (7.8-10.1)	9.9 (8.6-11.2)	10.2 (9.0-11.5)	<.001	
2.0-3.9	5.9 (5.1-6.7)	6.9 (6.1-7.8)	7.7 (6.8-8.6)	8.3 (7.2-9.5)	7.3 (6.3-8.2)	8.0 (6.9-9.2)	8.1 (7.1-9.0)	9.3 (8.4-10.3)	8.4 (7.5-9.4)	10.0 (8.6-11.3)	<.001	
≥4.0	6.2 (5.5-7.0)	6.8 (5.9-7.7)	6.7 (5.9-7.5)	7.4 (6.4-8.3)	7.3 (6.3-8.3)	7.8 (6.7-8.8)	7.8 (6.8-8.9)	9.3 (8.2-10.5)	8.8 (7.8-9.9)	9.4 (8.4-10.4)	<.001	
Missing	4.9 (4.0-5.8)	4.2 (3.6-4.9)	6.1 (5.2-7.0)	5.9 (5.1-6.8)	5.6 (4.6-6.6)	6.1 (5.0-7.1)	7.3 (6.1-8.4)	7.8 (6.5-9.2)	8.2 (6.8-9.5)	9.4 (8.0-10.8)	<.001	
Geographic region												
Northeast	5.5 (4.7-6.3)	5.1 (4.2-6.0)	7.3 (6.4-8.2)	6.5 (5.5-7.5)	6.3 (5.1-7.5)	7.2 (6.0-8.4)	9.2 (7.9-10.5)	8.3 (7.0-9.6)	9.5 (8.3-10.7)	10.3 (8.8-11.8)	<.001	<.001
Midwest	6.9 (6.1-7.6)	6.4 (5.6-7.2)	7.5 (6.6-8.4)	8.3 (7.2-9.3)	8.6 (7.5-9.7)	9.4 (8.1-10.7)	10.1 (8.8-11.3)	10.5 (9.3-11.7)	10.5 (9.4-11.6)	12.2 (10.8-13.6)	<.001	
South	6.6 (5.9-7.2)	8.1 (7.3-8.9)	8.4 (7.6-9.2)	8.0 (7.2-8.8)	8.7 (7.8-9.6)	9.5 (8.5-10.5)	10.7 (9.8-11.7)	11.5 (10.6-12.4)	11.0 (10.0-12.0)	11.1 (10.1-12.1)	<.001	
West	5.0 (4.2-5.9)	5.0 (4.2-5.7)	4.9 (4.1-5.8)	5.8 (5.0-6.7)	5.2 (4.4-5.9)	5.3 (4.5-6.1)	5.4 (4.5-6.3)	6.6 (5.7-7.5)	5.6 (4.8-6.4)	7.0 (6.1-7.8)	<.001	

Abbreviation: ADHD, attention-deficit/hyperactivity disorder.

^a^ Prevalence estimates were weighted.

^b^ *P* values for trends were calculated using weighted logistic regression models, which included survey cycle as a continuous variable and adjusted for age, sex, and race/ethnicity.

^c^ *P* values for interaction were calculated by including multiplicative terms of each stratum variable with survey cycle in the aforementioned logistic regression models.

^d^ The numbers of participants overall and with ADHD were unweighted.

## Discussion

In a nationally representative population, we estimated that the prevalence of diagnosed ADHD among US children and adolescents was 10.2% in 2016. The prevalence differed significantly by age, sex, race/ethnicity, family income, and geographic region. Similar to our findings, several previous studies also reported sex^8,9,10,11,12,13,14,19^ and racial/ethnic differences^15,24,25,26^ in the prevalence of ADHD.

Over the 20-year period from 1997 to 2016, we found a significant increase in the prevalence of diagnosed ADHD from 1997-1998 to 2015-2016. We found a consistent upward trend across subgroups by age, sex, race/ethnicity, family income, and geographic regions. The temporal trends in this study were consistent with previously reported trends in ADHD prevalence among US children and adolescents during earlier years.^8,9,10,11,12,13,14,15^ Taken together, these findings indicate a continuous increase in the prevalence of diagnosed ADHD among US children and adolescents. Previous studies conducted in the United Kingdom have also observed a significant increase in ADHD prevalence, although the prevalence estimates were substantially lower than those in the United States.^2^

Nonetiologic factors may partly explain the apparent increase in the prevalence of diagnosed ADHD in this study. Over the past 20 years, there have been expanded continuing medical education efforts about ADHD that enhanced physicians’ sensitivity to the diagnosis of ADHD. Changes in diagnostic criteria may also contribute to the increased number of children being diagnosed with ADHD.^7^ In particular, changes in the *Diagnostic and Statistical Manual of Mental Disorders* criteria that established the predominately inattentive presentation of ADHD led to significantly increased diagnosis in girls, who often fail to demonstrate classic hyperactive symptoms. In addition, increased public awareness, improved access to health services, and improved referral from primary care and communities to specialty mental health services may increase the likelihood of ADHD being identified on screening and diagnosis.^9^ Increased rates of diagnosed ADHD among black and Hispanic youths might reflect increased access to care and decreased stigma in those communities for receiving an ADHD diagnosis. The execution of the Affordable Care Act may also have increased access to care in lower socioeconomic status and minority groups. There is a common perception that ADHD is overdiagnosed in the United States, but this perception was not supported by scientific evidence based on review of prevalence studies and research on the diagnostic process.^27^

It remains to be understood how much of the observed apparent increase in diagnosed ADHD was attributed to etiologic factors; ADHD has a genetic component with an estimated heritability of 70% to 80%.^21^ In addition to genetic risk factors, environmental risk factors are believed to contribute to the development of ADHD.^1,28^ Prenatal and perinatal risk factors, including preterm birth, low birth weight, maternal cigarette smoking, and maternal use of certain medications or illicit substances during pregnancy, have been associated with ADHD risk.^29,30,31^ Attachment-related factors in early infancy have also been associated with ADHD in childhood.^32,33,34^ Environmental contamination, such as lead, organophosphate pesticides, and polychlorinated biphenyls exposure, during prenatal and/or postnatal periods is a possible risk factor for ADHD.^30^ In addition, nutritional deficiencies (eg, zinc, magnesium, and polyunsaturated fatty acids) may also be implicated in the development of ADHD.^1^ The contributions of these nongenetic and genetic risk factors to the etiologic source of ADHD, both separately and jointly, warrant further investigation.

### Strengths and Limitations

This study has several strengths. First, this study was based on a nationally representative sample of the US population, which facilitates the generalization of the findings to a broader population. Second, a large sample size with a multi-racial/ethnic diverse population was available, allowing us to assess disparities in ADHD prevalence according to population characteristics. Third, with a series of nationwide population-based cross-sectional surveys, we were able to evaluate the secular trends in ADHD prevalence over a period of observations as long as 20 years.

This study has some limitations. First, ADHD was ascertained by parent-reported physicians’ diagnosis, which may be subject to misreporting and recall bias. Second, we did not know whether the children and adolescents with a diagnosis of ADHD still had ADHD at the time of the survey across the survey years except in NHIS 2016. Previous studies have shown that the core symptoms of ADHD tend to decline with age, but inattentive features of ADHD are more likely to persist.^1^ In NHIS 2016, we found that 85% of children and adolescents with a history of ADHD diagnosis were reported as currently having ADHD, which was similar to the number reported in a previous study.^9^

## Conclusions

Among US children and adolescents aged 4 to 17 years, the estimated prevalence of diagnosed ADHD was 10.2% in 2015-2016, representing a significant increase in prevalence from 1997-1998. This continued upward trend in diagnosed ADHD among children and adolescents points to the need to better understand potentially modifiable environmental risk factors, as well as provide adequate resources for the diagnosis and treatment of affected individuals in the future.
